# Characterization of SARS-CoV-2-Specific Humoral and Cellular Immune Responses Induced by Inactivated COVID-19 Vaccines in a Real-World Setting

**DOI:** 10.3389/fimmu.2021.802858

**Published:** 2021-12-22

**Authors:** Ziwei Li, Tiandan Xiang, Boyun Liang, Hui Deng, Hua Wang, Xuemei Feng, Xufeng Quan, Xiaoyan Wang, Sumeng Li, Sihong Lu, Xuecheng Yang, Baoju Wang, Gennadiy Zelinskyy, Mirko Trilling, Kathrin Sutter, Mengji Lu, Ulf Dittmer, Dongliang Yang, Xin Zheng, Jia Liu

**Affiliations:** ^1^ Department of Infectious Diseases, Union Hospital, Tongji Medical College, Huazhong University of Science and Technology, Wuhan, China; ^2^ Joint International Laboratory of Infection and Immunity, Huazhong University of Science and Technology, Wuhan, China; ^3^ Institute for Virology, University Hospital of Essen, University of Duisburg-Essen, Essen, Germany

**Keywords:** COVID-19, SARS-CoV-2, inactivated vaccine, humoral immune responses, cellular immune responses

## Abstract

While the immunogenicity of inactivated vaccines against coronavirus disease 2019 (COVID‐19) has been characterized in several well-conducted clinical trials, real-world evidence concerning immune responses against severe acute respiratory syndrome coronavirus 2 (SARS‐CoV‐2) raised by such vaccines is currently missing. Here, we comprehensively characterized various parameters of SARS-CoV-2-specific cellular and humoral immune responses induced by inactivated COVID-19 vaccines in 126 individuals under real-world conditions. After two doses of vaccination, S-receptor binding domain IgG (S-RBD IgG) and neutralizing antibody (NAb) were detected in 87.06% (74/85) and 78.82% (67/85) of individuals, respectively. Female participants developed higher concentrations of S-RBD IgG and NAb compared to male vaccinees. Interestingly, a longer dosing interval between the first and second vaccination resulted in a better long-term SARS-CoV-2 S-RBD IgG response. The frequencies of CD4+ T cells that produce effector cytokines (IFN-γ, IL-2, and TNF-α) in response to stimulation with peptide pools corresponding to the SARS-CoV-2 spike (S), nucleocapsid (N) or membrane (M) protein were significantly higher in individuals received two doses of vaccine than those received one dose of vaccine and unvaccinated individuals. S, N, or M-specific CD4+ and CD8+ T cell responses were detectable in 95.83% (69/72) and 54.16% (39/72) of double-vaccinated individuals, respectively. The longitudinal analysis demonstrated that CD4+ T cell responses recognizing S, N, and M waned quickly after a single vaccine dose, but were boosted and became more sustained following a second dose. Overall, we provide a comprehensive characterization of immune responses induced by inactivated COVID-19 vaccines in real-world settings, suggesting that both humoral and cellular SARS-CoV-2-specific immunity are elicited in the majority of individuals after two doses of inactivated COVID-19 vaccines.

## Introduction

The coronavirus disease 2019 (COVID-19) pandemic caused by the severe acute respiratory syndrome coronavirus 2 (SARS‐CoV‐2) is an unprecedented burden to global healthcare systems and causes severe economic havoc e.g., through prolonged lockdowns. Effective COVID-19 vaccines already start to mitigate these problems – especially in regions with high vaccination coverage, suggesting that effective global vaccination has the potency to eventually terminate the COVID-19 pandemic. The astonishingly rapid implementation of COVID-19 vaccination programs is unprecedented in the history of vaccine development and application (1). As of August 2021, more than 5 billion doses of COVID-19 vaccines have been administered globally (2). This number comprises at least 18 different COVID-19 vaccines, which utilize a broad range of vaccine principles such as inactivated virus particles, mRNAs and viral vectors expressing the viral spike protein, or adjuvanted spike protein subunits (3). Inactivated vaccines belong to the most frequently used types of COVID-19 vaccines, and as of August 2021, over 2 billion doses of inactivated COVID-19 vaccines have been administered just in China (2, 4). The development of inactivated vaccines is a mature technology, which is widely used for the prevention and control of emerging infectious diseases (5). Inactivated vaccines are produced by growing SARS-CoV-2 in cell culture, usually on Vero cells, followed by chemical inactivation of the virus (6). Because the whole virus is presented to the immune system, immune responses are likely to target not only the spike protein of SARS-CoV-2 but also the matrix, envelope and nucleoprotein (7).

The effectiveness and immunogenicity of the 3 inactivated COVID-19 vaccines currently in use in China, namely “BBIBP-CorV”, “CoronaVac”, and “WIBP-CorV”, have been demonstrated in several clinical trials (5, 8–12). In each of these studies, standardized vaccination protocols were precisely followed, using inactivated vaccines from identical companies at well-defined intervals between first and second vaccination. Obviously, authentic real-world practice is more flexible, pragmatic, and diverse various combinations of inactivated vaccines from different companies are applied for the first and second vaccination, and the intervals between the two vaccinations vary to a certain degree. To our knowledge, data of SARS-CoV-2-specific humoral and cellular immune responses induced by inactivated COVID-19 vaccines in the real-world practice are not available so far. To generate such information, we recruited volunteers who received inactivated COVID-19 vaccines in real-world practice, and characterized their antibodies and T cell responses recognizing SARS-CoV-2. Our data suggest that both the humoral and the cellular SARS-CoV-2-specific immune responses are elicited in the majority of individuals after two doses of inactivated vaccines.

## Materials and Methods

### Study Design and Participants

Healthy adults, aged 19 to 79 years, without history of SARS-CoV-2 infection (*via* serological and nucleic acid test) were eligible for enrollment in the study. Exclusion criteria were as follows: confirmed natural SARS-CoV-2 infection; working in an environment posing a high risk for an exposure to SARS-CoV-2; symptoms indicating acute infections such as fever, cough, runny nose, sore throat, diarrhoea, dyspnoea, or tachypnoea during a 14 day period prior to sampling; abnormalities in laboratory tests; pregnancy or lactation; a history of autoimmune diseases; and prior or ongoing use of immunotherapy. All vaccinated participants and vaccinees immunized with one or tow doses of “BBIBP-CorV” (6.5 U per 0.5 ml of aluminium hydroxide per dose), or “CoronaVac”(600 SU per 0.5 ml of aluminium hydroxide per dose), or “WIBP-CorV” (200 WU per 0.5 ml of aluminium hydroxide per dose) were recruited at the Department of Infectious Diseases, Union Hospital, Tongji Medical College, Huazhong University of Science and Technology from December 2020 to July 2021. Informed written consent was obtained from each participant before sampling, and the study protocol was approved by the local medical ethics committee of Union Hospital, Tongji Medical College, Huazhong University of Science and Technology (2021-0570) and Chinese Clinical Trial Registry (ChiCTR2100048837). The study was conducted in accordance with the guidelines of the Declaration of Helsinki.

### Preparation of PBMCs

Peripheral blood mononuclear cells (PBMCs) were isolated using Ficoll density gradient centrifugation (DAKEWE Biotech, China) and rapidly assessed by flow cytometry analysis without intermittent cryo-preservation.

### Detection of SARS-CoV-2 Neutralizing Antibody and S-RBD IgG

Antibodies disturbing the binding of S to the ACE2 [for simplicity referred to as neutralizing antibody (NAb)], and IgG antibody recognizing the SARS-CoV-2 receptor binding domain (RBD) of the S protein (for simplicity refer to as S-RBD IgG) were tested by competitive or indirect chemiluminescence immunoassay (CLIA), respectively, by MAGLUMI™ 4000 Plus (Snibe, Shenzhen, China). The SARS-CoV-2 NAb assay described above is a qualitative detection of NAb in human serum and plasma, which is mainly used for the evaluation of NAb in patients recovering from COVID-19 or the auxiliary evaluation of the effect of the COVID-19 vaccines.

For the detection of SARS-CoV-2 NAb, in brief, human plasma, buffer, and magnetic microbeads coated with ACE2 antigen and ABEI labeled with recombinant SARS-CoV-2 S-RBD antigen are mixed thoroughly and incubated. SARS-CoV-2 NAb present in the plasma compete with ACE2 antigen immobilized on magnetic microbeads for binding recombinant SARS-CoV-2 S-RBD antigen labeled with ABEI. After precipitation in a magnetic field, chemiluminescent reaction were initiated and the light signal is measured by a photomultiplier as relative light units (RLUs), which is inversely proportional to the concentration of SARS-CoV-2 NAb presented in the plasma.

For the detection of S-RBD IgG, in brief, human plasma, buffer, and magnetic microbeads coated with S-RBD recombinant antigen are mixed thoroughly and incubated, forming immune-complexes. After precipitation in a magnetic field, ABEI labeled with anti-human IgG antibody were added to incubate to form complexes. Then, chemiluminescent reaction were initiated and the light signal is measured by a photomultiplier as RLUs, which is proportional to the concentration of S-RBD IgG presented in the plasma.

A study performed with the SARS-CoV-2 NAb and S-RBD IgG was obtained by testing 381 individuals neither SARS-CoV-2 infection nor vaccination, 99% SARS-CoV-2 NAb values were ≤ 0.05 μg/ml and 99% S-RBD IgG values were < 1.0 AU/ml. Cut-off value for SARS-CoV-2 NAb was 0.05 μg/ml and 1.0 AU/ml for S-RBD IgG.

### Analysis of Effector T Cell Responses

Three pools of lyophilized peptides, consisting mainly of 15-mer sequences with 11 amino acids (aa) overlap, that cover the entire sequences of the surface glycoprotein (S, Cat No.RP30027, Genscript Biotech Corporation, Nanjing, China), the nucleocapsid phosphoprotein (N, Cat No. RP30013, Genscript Biotech Corporation, Nanjing, China) or the membrane glycoprotein (M, Cat No. 30022, Genscript Biotech Corporation, Nanjing, China) of SARS-CoV-2, were used for cell stimulation. On day 1, PBMCs were cultivated in complete medium [RPMI 1640 containing 10% (v/v) fetal calf serum, 100 U/ml penicillin, 100 μg/ml streptomycin, and 100 μM 4-(2-hydroxyethyl)-1-piperazine ethanesulfonic acid (HEPES) buffer] with recombinant interleukin (IL)-2 (20 U/ml; Hoffmann-La Roche, Italy). Cells without anti-CD3, anti-CD28 and peptide stimulation served as negative control (unstimulated control). Cells with anti-CD3 (1 μg/ml; Invitrogen, USA) and anti-CD28 (1 μg/ml; Invitrogen, USA) stimulation served as positive control. Cells stimulated with S, N, or M peptide pools (1 μg/ml) in the presence of anti-CD28 served as peptide stimulation groups. Fresh medium containing IL-2 was added on day 4 and 7. On day 10, cells were restimulated for 5 hours with the same peptide pool in the presence of brefeldin A (BD Biosciences, San Diego, CA). Cells previously stimulated by anti-CD3 and anti-CD28 were restimiulated by phorbol 12-myristate 13-acetate (PMA) and ionomycin (Iono) for 5 hours served as positive controls. Cells were then tested for IFN-γ, IL-2, and TNF-α expression by intracellular cytokine staining. Specific cytokine responses were calculated by subtracting the background activation i.e. the percentage of cytokine positive cells in the unstimulated control. T cell responses were defined as being detectable in the case that the frequency in specifically stimulated cultures exceeded the unstimulated control at least two-fold (stimulation index > 2). Samples with responseless positive controls were excluded from further analysis.

### Flow Cytometry

Surface and intracellular staining for flow cytometry analysis were performed as described previously (13, 14). For surface staining, cells were incubated with relevant fluorochrome-labeled antibodies (eFluor 780-anti-CD3, PE-Cy7-anti-CD8, and PerCP-Cy5.5-anti-CD4) for 30 min at 4°C in the dark. For intracellular cytokine staining, cells were fixed and permeabilized using the Intracellular Fixation & Permeabilization Buffer Set (Invitrogen, USA) and subsequently stained with FITC-anti-IFN-γ, PE-anti-IL-2 and APC-anti-TNF-α (Invitrogen, USA). Approximately 100,000 PBMCs were acquired for each sample using a BD FACS Canto II flow cytometer. Data analysis was performed using the FlowJo software V10.0.7 (Tree Star, Ashland, OR, USA). Cell debris and dead cells were excluded from the analysis based on scatter signals and Fixable Viability Dye eFluor 506.

### Statistical Analysis

Statistical analyses were performed using the SPSS statistical software package (version 22.0, SPSS Inc., Chicago, IL, USA). The Shapiro-Wilk method was used to test for normality. Mann-Whitney U-test, one-way ANOVA and Pearson product-moment correlation coefficient were used where appropriate. All reported *P* values were two-sided, and a P value below 0.05 was considered as hallmark of statistical significance (*,*P*<0.05; **, *P <*0.01; ***, *P <*0.001; ****, *P <*0.0001).

### Data Availability

We support data sharing of the individual participant data. The individual participant data that underlie the results reported in this Article, after deidentification will be shared. Researchers who provide a scientifically sound proposal will be allowed to access the de-identified individual participants data. Proposal should be sent to the corresponding authors, at xinz@hust.edu.cn or jialiu77@hust.edu.cn. The proposals will be reviewed and approved by the funders, investigator and collaborators on the basis of scientific merit. To gain access, data requestors will need to sign a data access agreement.

## Results

### Characterization of the Study Cohort

The vaccine-induced SARS-CoV-2-specific humoral and cellular immune responses were characterized in 168 blood samples collected from 126 healthy individuals, among which 32 samples were collected prior to vaccination and 136 samples were collected after inoculation with inactivated COVID-19 vaccines (BBIBP-CorV, CoronaVac, and/or WIBP-CorV). The demographic profiles of all participants are shown in **Table 1**. The age of participants received two doses of vaccines was older than the individuals received single dose, and no difference was observed in sex of the three groups. The dosing interval between the first and second vaccination was 21-63 days (median: 43 days). In 104 participants, blood samples were collected at a single time point before or after vaccination. In 22 participants, blood samples were collected at 2 to 4 different time points before and after the first and second vaccination. The interval time between the blood donation and the first and second vaccination was 10-45 days (median: 29 days) and 10-57 days (median: 30 days), respectively. In the first and second vaccination, 22.97% (17/74) of the participants were inoculated with vaccines from the same manufacturer (5.41%, 4/74, BBIBP-CorV; 4.05%, 3/74, CoronaVac; 13.52%, 10/74, WIBP-CorV), and 77.03% (57/74) of the vaccinees were immunized with vaccines of different manufactures (10.81%, 8/74, BBIBP-CorV+ CoronaVac; 20.27%, 15/74, BBIBP-CorV+ WIBP-CorV; 45.95%, 34/74, CoronaVac+ WIBP-CorV). Overall adverse reactions were reported in 34.26% (34/101) of the participants within 7 days of each injection, among which the most common were injection site pain (30.69%, 31/101), followed by induration (0.99%, 1/101), fever (0.99%, 1/101), muscle pain (1.98%, 2/101), fatigue (0.99%, 1/101), and headache (0.99%, 1/101). No serious adverse reactions occurred.

**Table 1:** Baseline characteristics of the cohort.

Characteristic	Baseline	After 1^st^ vaccination	After 2^nd^ vaccination		*P* value
**No. of participants**	32	41	85		NA
**Sex (Male/Female)**	10/22	15/26	29/59		NS^*^
**Age, median, year-old**	28 (21-79)	27 (22-55)	34 (19-77)		0.02^#^
**Manufacturer information of each vaccination**					NA
			1^st^ vaccination	2^nd^ vaccination	
BBIBP-CorV		5.00% (2/40)	17.33% (13/75)	25.68% (19/74)	
CoronaVac		22.50% (9/40)	10.67% (8/75)	54.05% (40/74)	
WIBP-CorV		72.50% (29/40)	72.00% (54/75)	20.27% (15/74)	
**Manufacturer information of the two vaccinations**					NA
Single manufacturer			22.97% (17/74)		
BBIBP-CorV			5.41% (4/74)		
CoronaVac			4.05% (3/74)		
WIBP-CorV			13.51% (10/74)		
Mixed manufacturers			77.03% (57/74)		
BBIBP-CorV+ CoronaVac			10.81% (8/74)		
BBIBP-CorV+ WIBP-CorV			20.27% (15/74)		
CoronaVac+ WIBP-CorV			45.95% (34/74)		
**Overall adverse reactions**		34.26% (34/101)			NA
Injection site adverse reactions					
Pain		30.69% (31/101)			
Redness and swelling		0			
Itch		0			
Induration		0.99% (1/101)			
Systemic adverse reactions					
Fever		0.99% (1/101)			
Fatigue		0.99% (1/101)			
Somnolence		0			
Headache		0.99% (1/101)			
Muscle pain		1.98% (2/101)			
Rash		0			
Vomiting		0			
Diarrhea		0.99% (1/101)			
**Underlying diseases**					NA
Hypertension	3.13% (1/32)	0	2.70% (2/74)		
Diabetes	0	2.44% (1/41)	2.70% (2/74)		
Cardiovascular diseases	0	0	1.35% (1/74)		
COPD	0	0	0		
Tumor	0	0	0		
Others	0	4.88% (2/41)	2.70% (2/74)		
**Sampling times**					NA
1	82.54% (104/126)				
2	7.94% (10/126)				
3	3.17% (4/126)				
4	6.35% (8/126)				

*Chi-square test was used to test the statistical significance.

^#^One-way ANOVA followed by Turkey’s multiple comparisons test was used to test the statistical significance. COPD, chronic obstructive pulmonary disease; NA, not available; NS, not significant.

### Characterization of Vaccine-Induced Antibody and T Cell Responses Specific to SARS-CoV-2

**Figure 1:**
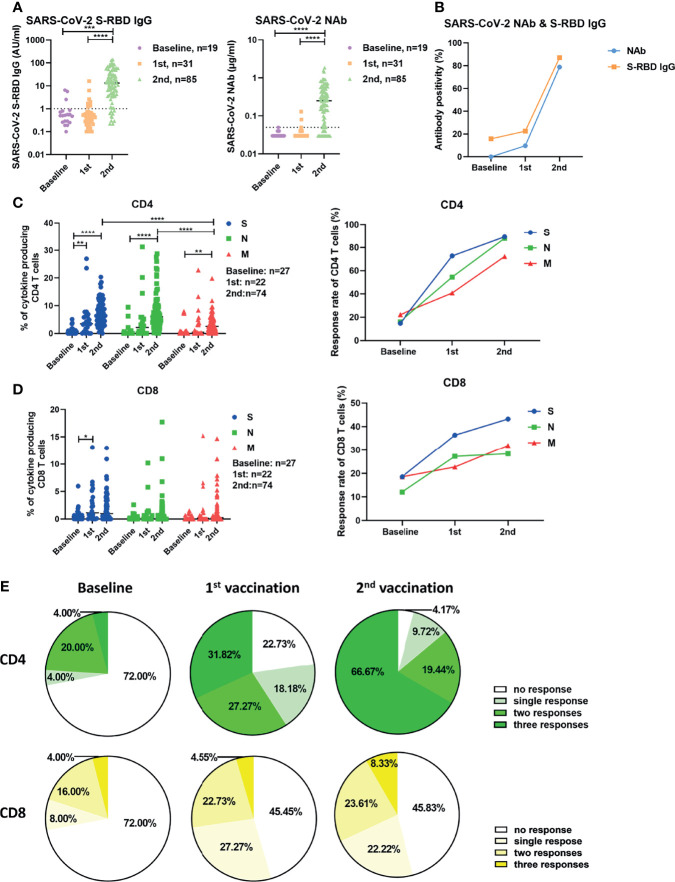
Characterization of SARS-CoV-2-specific antibodies and T cell responses in individuals before and after vaccination. **(A)** SARS-CoV-2 S-RBD IgG and NAb levels in serum before and after vaccination. The dotted line indicates the cut-off value of the antibody. **(B)** The antibody positivity for SARS-CoV-2 S-RBD IgG and NAb before and after vaccination. **(C)** The magnitude and response rate of overall cytokine responses of CD4+ T cells against S, N, and M of SARS-CoV-2 in the participants before and after the first and second vaccination. **(D)** The magnitude and response rate of overall cytokine responses of CD8+ T cells against S, N, and M of SARS-CoV-2 in the participants pre- and post-vaccination. **(E)**The breadth of CD4+ and CD8+ T cell responses before and after the first and second vaccination. Baseline: before vaccination; 1st: after the first vaccination; 2nd: after the second vaccination. Each symbol represents an individual donor with a line indicating the median of each group. One-way ANOVA followed by Turkey’s multiple comparisons test was used to test the statistical significance of data shown in **(A)**, **(C, D)**. *P < 0.05; **P < 0.01; ***P < 0.001; ****P < 0.0001.

Firstly, vaccine-induced humoral responses were characterized by measuring serum SARS-CoV-2 S-RBD IgG and neutralizing antibody (NAb) concentrations. At baseline, none of the participants had detectable neutralizing antibodies. Concentrations of S-RBD-specific IgG were significantly higher after the second vaccination (median: 13.3 AU/ml) compared to baseline levels (0.5 AU/ml) (**Figure 1A**). The concentrations of NAb were significantly higher after the second vaccination (median: 0.25 μg/ml) compared to the baseline (median: 0.03 μg/ml) and compared to the response after the first vaccination (median: 0.03 μg/ml) (**Figure 1A**). The seropositivity rates of S-RBD-specific IgG at baseline, after the first and the second vaccination, were 15.79% (3/19), 22.58% (7/31) and 87.06% (74/85), respectively (**Figure 1B**). The seroconversion rates regarding NAb after the first and the second vaccination were 9.68% (3/31) and 78.82% (67/85), respectively (**Figure 1B**).

Next, we examined the vaccine-induced SARS-CoV-2-specific cellular immunity by stimulating fresh isolated PBMCs with 3 panels of overlapping peptides spanning the SARS-CoV-2 proteins S, N, and M. We used an intracellular cytokine staining flow cytometry assay (**Figure S1**), and the magnitude of overall cytokine responses (IFN-γ, IL-2, and TNF-α) for CD4+ and CD8+ T cells for all participants are shown in **Figure 1C, D**. The frequencies of effector cytokine-producing CD4+ T cells in response to S, N, or M peptide pool stimulation were significantly higher after the second vaccination compared with the baseline responses and after the first vaccination (**Figure 1C**). The intensities of CD4+ T cell responses against S and N were significantly higher compared to those against M after two doses of vaccination (**Figure 1C**, left). In contrast to the observation with CD4+ T cells, the first dose of vaccination only induced a significant increase in the intensity of S-specific CD8+ T cell responses in participants (**Figure 1D**, left). The S-, N-, and M-specific CD8+ T cell responses were detected in 18.52% (5/27), 12.00% (3/25), and 18.52% (5/27) of participants at baseline, respectively (**Figure 1D**, right). These rates reached 36.36% (8/22), 27.27% (6/22), and 22.73% (5/22) after the first vaccination, but did not further increase after the second vaccination (**Figure 1D**, right). No significant differences were observed between the intensities of CD8+ T cell responses against S, N, and M (**Figure 1D**, left). Similar results were observed when the intensities of T cell responses were measured by single effector cytokine (IFN-γ, IL-2, or TNF-α) expression (**Figure S2**). Additionally, we analyzed the breadth (to how many peptide pools the T cells responded) of SARS-CoV-2-specific T cell responses induced by vaccination. At baseline, CD4+ T cell responses against a single, two, or three peptide pools of the different proteins were detected in 4.00% (1/25), 20.00% (5/25), and 4.00% (1/25) of participants, respectively (**Figure 1E** **, left**). These ratios were 18.18% (4/22), 27.27% (6/22), and 31.82% (7/22) in participants who received the first vaccination (**Figure 1E**, middle), and reached 9.72% (7/72), 19.44% (14/72), and 66.67% (48/72) in participants who received the second vaccination, respectively (**Figure 1E**, right). There were only 4.17% (3/72) of participants, who did not mount detectable CD4+ T cell responses after the second vaccination (**Figure 1E**, right). At the baseline, CD8+ T cell responses against a single, two, or three peptide pools of the different viral proteins were detected in 8.00% (2/25), 16.00% (4/25), and 4.00% (1/25) of participants, respectively (**Figure 1E**, left). These ratios were 27.27% (6/22), 22.73% (5/22), and 4.55% (1/22) in participants following the first vaccination (**Figure 1E**, middle), and were 22.22% (16/72), 23.61% (17/72), and 8.33% (6/72) in participants who received the second vaccination (**Figure 1E**, right). However, there were 45.83% (33/72) of participants, who showed no detectable CD8+ T cell responses after the second vaccination (**Figure 1E**, right).

Next, we explored whether the intensities of vaccine-induced humoral and cellular immune responses were correlated. In general, there were no significant correlations between the intensities of S-, N-, or M-specific CD4+ or CD8+ T cell responses and the serum concentrations of S-RBD IgG or NAb after the first or second vaccination (**Figures S3A–D**). We only observed that the intensity of S-specific CD8+ T cell response was very weakly, but borderline statistical significantly, correlated with the concentrations of NAb after two doses of vaccination (**Figure S3D**).

Taken together, these data suggest that two doses of inactivated vaccine elicit SARS-CoV-2-specific antibodies and CD4+ T cell responses in most individuals.

### Influences of Sex and Age on Vaccine-Induced SARS-CoV-2-Specific Antibody and T Cell Responses

**Figure 2:**
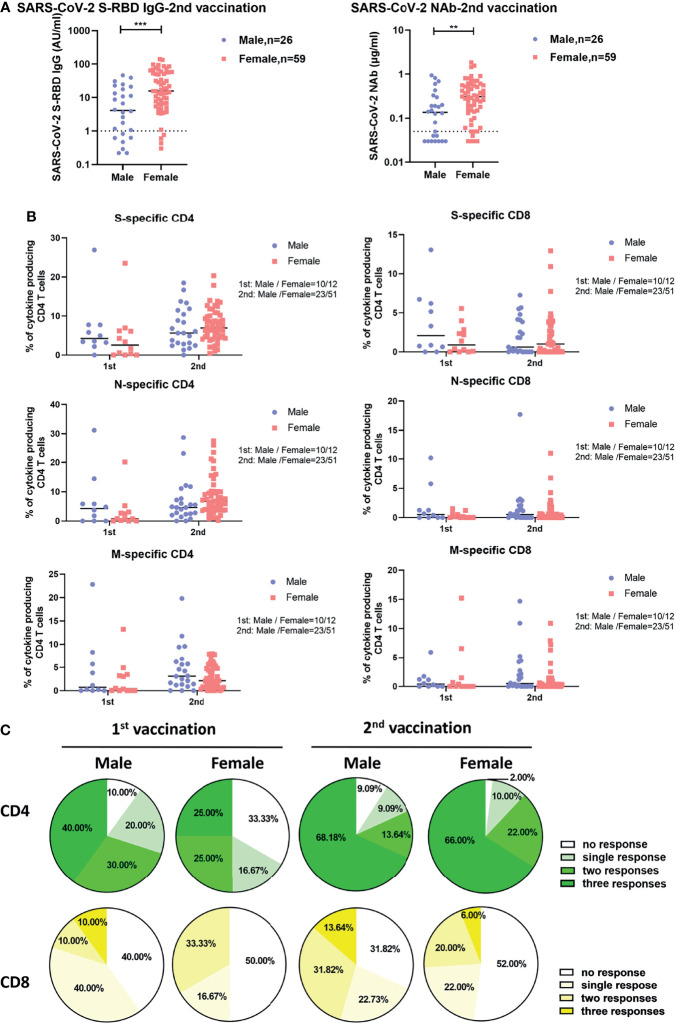
Differences in SARS-CoV-2-specific humoral and cellular immune responses between male and female vaccinees. **(A)** Comparison of the SARS-CoV-2 S-RBD IgG and NAb levels in serum after the second vaccination between male and female vaccinees. The dotted line indicates the cut-off value of the antibody. **(B)** Comparison of the magnitude of CD4+ and CD8+ T cell responses against S, N, and M of SARS-CoV-2 between male and female vaccinees. **(C)** Comparison of the breadth of CD4+ and CD8+ T cell responses between male and female vaccinees. Each symbol represents an individual donor with a line indicating the median of each group. Mann Whitney U test was used to test the statistical significance of data shown in **(A, B)**. **P < 0.01; ***P < 0.001.

We next explored whether sex and age influence the intensities of vaccine-induced SARS-CoV-2-specific antibody and T cell responses. As shown in **Figure 2A**, after two doses of inactivated vaccine, female participants showed significantly higher concentrations of SARS-CoV-2-specific S-RBD IgG and NAb than male participants did. No significant differences in the intensities of both CD4+ and CD8+ T cell responses were observed between females and males irrespective of the number of applied vaccine doses (**Figure 2B**). After two doses of vaccine, SARS-CoV-2-specific CD4+ T cell responses were detectable in 98.00% of female participants versus 90.91% of male participants (**Figure 2C**). Moreover, we also examined potential correlations between age and the intensities of vaccine-induced SARS-CoV-2-specific antibody and T cell responses. As shown in **Figure S4**, we only observed that the breadth of CD8+ T cell responses was negatively correlated with age after the first vaccination (**Figure S4B**), while no obvious correlations between age and the concentrations of SARS-CoV-2 S-RBD IgG and NAb, or the intensities of S-, N-, and M-specific CD4+ and CD8+ T cell responses were observed after the second vaccination.

### Correlation Between Vaccine-Induced Immune Responses and the Time Post Vaccination

**Figure 3:**
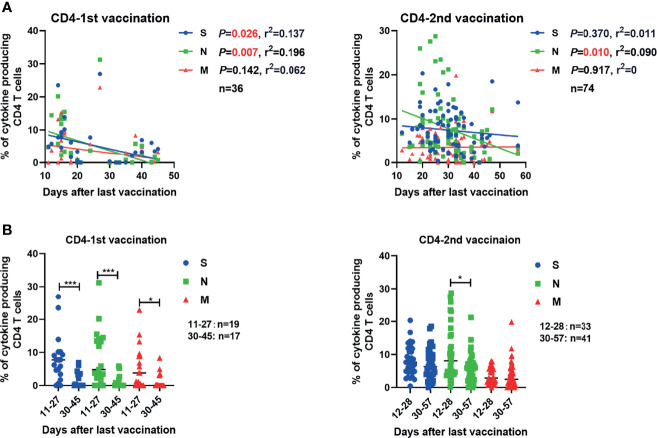
Correlation between the SARS-CoV-2-specific T cell responses and the time that had elapsed from the last vaccination. **(A)** The correlations between SARS-CoV-2-specific CD4+ T cell responses and days after the first and second vaccination are shown. **(B)** Comparison of the magnitudes of SARS-CoV-2-specific CD4+ and CD8+ T cell responses before and after 30 days after the first or second vaccination. Pearson product-moment correlation coefficient test was used to test the significance of data shown in **(A)** and P value and r^2^ value (correlation coefficient) are indicated in each panel. Each symbol represents an individual donor with a line indicating the median of each group. Mann Whitney U test was used to test the statistical significance of data shown in **(B)**. *P < 0.05; ***,*P < *0.001.

Next, we analyzed how the intensities of vaccine-induced SARS-CoV-2-specific antibody and T cell responses change over time post vaccination. The concentrations of serum S-RBD IgG and NAb showed no significant correlation with the time after the second vaccination up to 57 days (**Figure S5A**). The frequencies of effector cytokine-producing CD4+ T cells in response to S (r^2 ^= 0.137, *P*=0.026) and N (r^2 ^= 0.196, *P*=0.007) peptide pool stimulation were significantly inversely correlated with the days post the first vaccination (**Figure 3A**). After the second vaccination, we observed that only the frequency of effector cytokine-producing CD4+ T cells in response to N (r^2 ^= 0.090, *P*=0.010) stimulation was negatively correlated with days post the second vaccination (**Figure 3A**). Like for CD4+ T cell responses, the frequency of effector cytokine-producing CD8+ T cells in response to N (r^2 ^= 0.142, *P*=0.024) peptide pool stimulation was significantly inversely correlated with the days post the first vaccination (**Figure S5B**, left). In contrast, no significant negative correlation between the intensities of CD8+ T cell responses and the time post the second vaccination was observed (**Figure S5B**, right). No significant correlation between the breadth of SARS-CoV-2 T cell responses and the days post the first or second dose of vaccine was observed (**Figure S5C**). Similar results were observed when the magnitude of T cell responses was analyzed according to single effector cytokine (IFN-γ, IL-2, or TNF-α) expression levels (**Figure S6**). We next stratified the participants into two groups regarding to the time post their last vaccination: more or less than 30 days. We observed that the intensities of CD4+ T cell responses against S, N, and M were significantly lower in participants who received the first dose of vaccine more than 30 days before as compared to those individuals who got it less than 30 days ago (**Figure 3B**). After the second vaccination, only the intensities of CD4+ responses against N were significantly lower in participants who received the second dose of vaccine over 30 days compared to those with less than 30 days (**Figure 3B**). Taken together, these results indicated that the SARS-CoV-2-specific CD4+ T cell responses induced by a single dose of inactivated COVID-19 vaccine were short-lived, but can be strengthened and perpetuated by the booster vaccination.

### Kinetics of Vaccine-Induced SARS-CoV-2-Specific Antibody and T Cell Immune Responses

**Figure 4:**
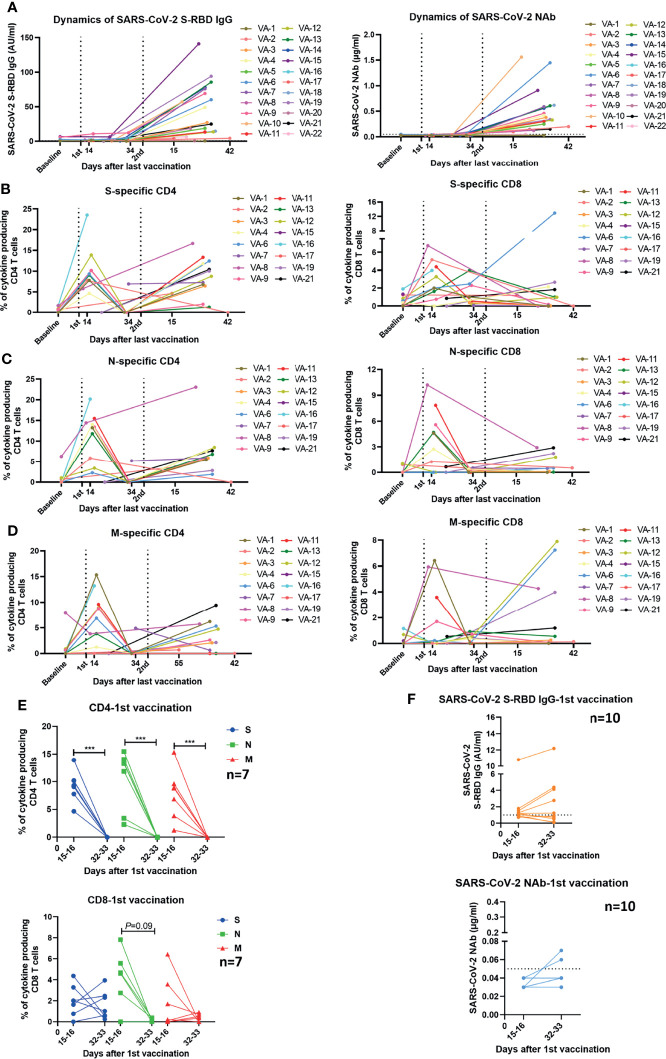
Kinetics of humoral and cellular immune responses in individuals who received vaccines. **(A)** Dynamic changes of SARS-CoV-2 S-RBD IgG and NAb levels in 22 vaccinees. The dotted line indicates the cut-off value of the antibody. **(B–D)** Dynamic changes of the magnitude of T cell responses against S, N, and M in 16 vaccinees. The dynamics of different individuals are exhibited by lines of different colors. The numbers noted under the X-axis demonstrate the starting and ending days post the last vaccination. **(E)** Comparison of the magnitudes of T cell responses in 7 vaccinees detected at different time points after the first vaccination. **(F)** Comparison of the SARS-CoV-2 S-RBD IgG and NAb levels in 10 vaccinees at different time points after the first vaccination. The dotted line indicates the cut-off value of the antibody. Baseline: before vaccination; 1st: after the first vaccination; 2nd: after the second vaccination. Each symbol represents an individual donor with a line indicating the median of each group. Mann Whitney U test was used to test the statistical significance of data shown in **(E, F)**. ***P < 0.001.

We further assessed the longitudinal changes of SARS-CoV-2-specific S-RBD IgG and NAb concentrations, as well as the intensities of SARS-CoV-2-specific T cell responses in 22 participants who had been sampled 2-4 times during the observation period. 2 of the 22 participants received vaccines of the same manufacturer (WIBP-CorV, VA-2 and VA-22) and the other 20 participants were inoculated with vaccines from different manufacturers (WIBP-CorV+ CoronaVac, VA-1, VA-3~VA-7, VA-9, VA-10, VA-12~VA-16, VA-18~VA-21; WIBP-CorV+ BBIBP-CorV, VA-8, VA-11, VA-17). In agreement with the aforementioned results of our cross-sectional analysis, the longitudinal analysis demonstrated that the second vaccination was required to increase the serum concentrations of SARS-CoV-2-specific S-RBD IgG and NAb (**Figure 4A**). Increased intensities of S-, N-, and M-specific CD4+ T cell responses were observed in the majority of participants two weeks after the first vaccination, however, these responses quickly decreased to undetectable levels 2-3 weeks later (**Figures 4B–D**). The first vaccination induced increased intensities of S-, N-, and M-specific CD8+ T cell responses in some participants two weeks after the vaccination. Similar to the CD4+ T cells, the CD8+ T cell responses also started to decline shortly afterwards (**Figures 4B–D**). In contrast to the observation that T cell responses decreased significantly at the later time point after a single dose of vaccination (**Figure 4E**), concentrations of SARS-CoV-2-specific S-RBD IgG and NAb started to increase slightly at the later time point in some of the participants (**Figure 4F**). Nevertheless, increased S-RBD IgG and NAb responses, as well as SARS-CoV-2-specific CD4+ T cell responses were observed in the majority of participants after the second vaccination compared to the baseline (**Figures 4A–D**). Taken together, the results of the longitudinal analysis further underlined the importance of boost vaccinations for generating effective and sustained humoral and cellular immunity against SARS-CoV-2.

### Influence of the Dosing Interval on Vaccine-Induced SARS-CoV-2-Specific Antibody and T Cell Immune Responses

**Figure 5:**
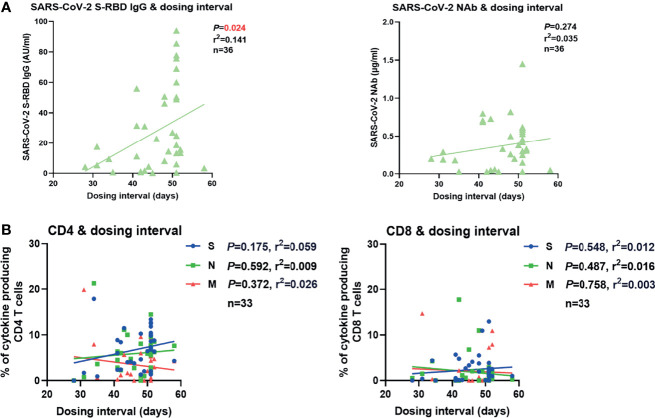
Correlation between SARS-CoV-2-specific humoral and cellular immune responses and dosing interval. **(A)** Correlation between the SARS-CoV-2 S-RBD IgG (left) and NAb (right) levels 4-6 weeks after the second vaccination and the days of dosing interval. **(B)** Correlation between the magnitudes of SARS-CoV-2-specific CD4+ (left) and CD8+ (right) T cell responses 4-6 weeks after the second vaccination and the days of dosing interval. Pearson product-moment correlation coefficient test was used to test the significance and P value and r^2^ value (correlation coefficient) are indicated in each panel.

In our real-world study, the dosing interval between the first and second vaccination varied greatly (21-63 days). Therefore, we examined whether the dosing interval had an impact on vaccine-induced SARS-CoV-2-specific antibody and T cell immune responses. No significant correlation between the intensities of antibodies or T cell responses after the second vaccination and the days of dosing interval was observed in overall participants (**Figure S7A**). Since the time that elapsed from the last vaccination may also influence the intensity of SARS-CoV-2-specific immunity, we next stratified the participants into two groups according to the time post the second vaccination (2-4 weeks and 4-6 weeks). No significant correlation between the intensities of antibody or T cell responses after the second vaccination and the length of the dosing interval was observed in the participants of 2- to 4-week group (**Figure S7B**). However, a significantly positive correlation between the concentration of SARS-CoV-2 S-RBD IgG and the dosing interval was observed in the participants of the 4-6-week group (**Figure 5A**). No significant correlation between the concentration of NAb or the intensities of T cell responses and the days of dosing interval was observed in this group (**Figures 5A, B** **)**. Taken together, these results suggested that a longer dosing interval might favor better long-term SARS-CoV-2 S-RBD IgG responses after the second vaccination.

## Discussion

The establishment of antigen-specific T- and B-cell responses is essential for sustained protection against viral diseases. NAbs generated by B cells are able to bind to SARS-CoV-2 and directly interfere with viral entry into target cells, whereas T cell responses are thought to limit viral replication by diminishing the number of infected cells, and reduce COVID-19 severity (15). Here we provide, to our knowledge, the first analysis of SARS-CoV-2-specific antibody and T cell responses in a cohort of vaccinees who received inactivated COVID-19 vaccines in real-world settings. We show that seroconversion rates concerning S-RBD IgG and NAb were 87.06% and 78.82%, respectively, at 10-57 days post the second dose of vaccination. In previous phase 1/2 clinical trials that assessed inactivated COVID-19 vaccines, seroconversion rates of S-RBD IgG and NAb ranged from 83% to 100% and from 25 to 100%, respectively (8–11). The dosing interval seems to be an important factor that influences the seroconversion rates of NAb and S-RBD IgG since in the phase 1 clinical trials of CoronaVac, the 28-day-interval group showed higher seroconversion rates of NAb and S-RBD IgG than the 14-day-interval group (9). It was also reported that extension of the interval between vaccine doses for the BNT162b2 mRNA vaccine from the conventional 3-4 week regimen to 6-14 weeks resulted in higher NAb levels (16). This is consistent with our findings in the real-world settings that a longer interval between the first and second vaccination results in higher concentrations of S-RBD IgG 4 weeks after the second vaccination. Moreover, we also observed that females showed superior antibody responses than males after receiving two doses of inactivated vaccines. Interestingly, a trend for more robust SARS-CoV-2-specific humoral responses in females was also observed in individuals who received anti-COVID-19 mRNA vaccines (17, 18). It is known that females generally exhibit greater humoral and cell-mediated immune responses to antigenic stimulation, vaccination, and infection than males do (19–21). In line with our current observation, a very recent study reported that the efficacy of inactivated vaccines against COVID-19 was higher in females than in males during the during the outbreak of the Delta variant in May 2021 in Guangzhou city, China (22).

So far, the virus-specific T cell immunity induced by inactivated COVID-19 vaccines is far from being well defined. Yao Deng et al. recently reported that BBIBP-CorV recipients raise specific T cell responses that recognize multiple structural proteins (S, N, and E proteins) of SARS-CoV-2 (23). However, the study only enrolled 10 healthy individuals and T cell responses were only characterized by ELISpot, which does not allow distinguishing CD4+ and CD8+ T cell responses. Here, we analyzed the SARS-CoV-2-specific T cell responses induced by inactivated COVID-19 vaccines in more detail and more comprehensively. We show that the magnitude of SARS-CoV-2-specific CD4+ T cell responses was already significantly increased after a single dose vaccination and further increased after the second administration. The SARS-CoV-2-specific CD4+ T cell responses were detectable in over 95% of participants after two doses and S- and N-specific CD4+ T cell responses were significantly stronger than M-specific CD4+ T cell responses. Similarly, it has been reported that in COVID-19 patients the CD4 T cell responses to S are the most abundantly detected responses, followed by the responses to N and M (24–26).Corresponding mechanisms should be addressed in future studies. However, SARS-CoV-2-specific CD8+ T cell responses induced by inactivated vaccines were rather weak and less frequently observed in the participants compared to the aforementioned CD4+ T cell responses. This finding is in accordance with our expectations since inactivated vaccines tend not to induce strong CD8+ T cell responses (9).

Another key issue that needs to be characterized is the duration of humoral and cellular immunity generated by inactivated vaccines. Our data suggest that virus-specific CD4+ T cell responses generated by single-dose vaccinations are extremely short-lived and last less than 5 weeks. A boost vaccination generates more robust S-, N-, and M-specific CD4+ T cell responses, which in most individuals lasted at least up to 2 months post the second administration. However, the intensities of SARS-CoV-2-specific CD4+ T cell responses induced by two doses of vaccination were still only one-third of the responses that we detected in convalescent individuals after COVID-19. Moreover, we observe that the intensity of N-specific CD4+ T cell response is negatively correlated with the time post the second vaccination, suggesting vaccine-induced cellular immune responses may already start to wane during our short observation period. This is in line with our recent finding that SARS-CoV-2-specific memory T cell responses in convalescent individuals are not long-lasting and wane profoundly 10 months after infection (data in submission). In this regard, our results argue in favor of booster immunizations of inactivated vaccines in order to maintain effective and lasting immunity including CD4+ T cells against SARS-CoV-2 infections.

There are several limitations in the current study. First, although the local spread of SARS-CoV-2 in Wuhan and the surrounding areas was prevented by strict non-pharmacologic intervention methods during the period in which the study was conducted, it is still difficult to absolutely exclude the pre-existing SARS-CoV-2-specific immunity in vaccinees induced by virus contact (e.g. by household contacts of asymptomatically infected persons or before initiation of the intervention methods). Second, other regions outside of S-RBD could also be neutralizing, however, only SARS-CoV-2 NAb and S-RBD IgG were detected in the current study. SARS-CoV-2-specific antibodies such as anti-S IgG and anti-N IgG should also be characterized in future study. Third, SARS-CoV-2-specific T cell responses targeting other structural and accessory proteins such as envelope (E) protein and open reading frame (ORF) 3,6,7 and 8 (27) were not characterized in the current study.

Collectively, we provide a comprehensive characterization of the immune responses induced by inactivated COVID-19 vaccines in real-world settings. While a single vaccination was insufficient to induce robust immune responses, both humoral and cellular SARS-CoV-2-specific immunity could be elicited in the majority of individuals who received two inactivated COVID-19 vaccine doses.

## Data Availability Statement

The raw data supporting the conclusions of this article will be made available by the authors, without undue reservation.

## Ethics Statement

The studies involving human participants were reviewed and approved by The local medical ethics committee of Union Hospital, Tongji Medical College, Huazhong University of Science and Technology. The participants provided their written informed consent to participate in this study.

## Author Contributions

ZL, TX, BL, XZ, and JL planned the experiment. ZL, TX, and BL performed the detection of T cell responses, clinical data collection and data analysis. HW conducted the detection of SARS-CoV-2 NAb and S-RBD IgG and XQ helped with the data collection of the antibodies. XF involved in the collection of blood samples. ZL and JL wrote the manuscript. HD, XW, SML, SHL, XY, BW, GZ, MT, KS, ML, UD, DY, and XZ revised the manuscript. All authors contributed to the article and approved the submitted version.

## Funding

This work is supported by the Fundamental Research Funds for the Central Universities (2020kfyXGYJ028, 2020kfyXGYJ046 and 2020kfyXGYJ016), the National Natural Science Foundation of China (81861138044 and 91742114), the National Science and Technology Major Project (2017ZX10202203-007-006, 2017ZX10202202-001-009, 2017ZX10202202-002-008, 2017ZX10202201-002-003), Science and Technology Key Project on Novel Coronavirus Pneumonia, Hubei Province (2020FCA002), the Deutsche Forschungsgemeinschaft (DI 714/22-1, ZE 893/2-1, RTG1949/2), the Medical Faculty of the University of Duisburg-Essen and Stiftung Universiätsmedizin, University Hospital Essen, Germany, and the Tongji-Rongcheng Center for Biomedicine, Huazhong University of Science and Technology.

## Conflict of Interest

The authors declare that the research was conducted in the absence of any commercial or financial relationships that could be construed as a potential conflict of interest.

## Publisher’s Note

All claims expressed in this article are solely those of the authors and do not necessarily represent those of their affiliated organizations, or those of the publisher, the editors and the reviewers. Any product that may be evaluated in this article, or claim that may be made by its manufacturer, is not guaranteed or endorsed by the publisher.
