# Supplementary material for: Characterization of SARS-CoV-2-Specific Humoral and Cellular Immune Responses Induced by Inactivated COVID-19 Vaccines in a Real-World Setting
Source: Front Immunol. 2021 Dec 22;12:802858. doi: 10.3389/fimmu.2021.802858 (PMC8727357; doi:10.3389/fimmu.2021.802858)
Supplement: Supplementary file 1 [file DataSheet_1.docx]

**
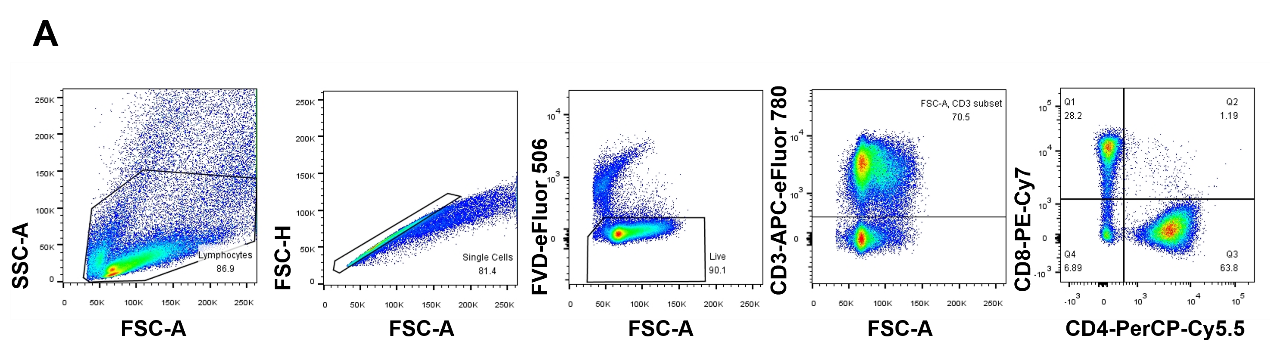

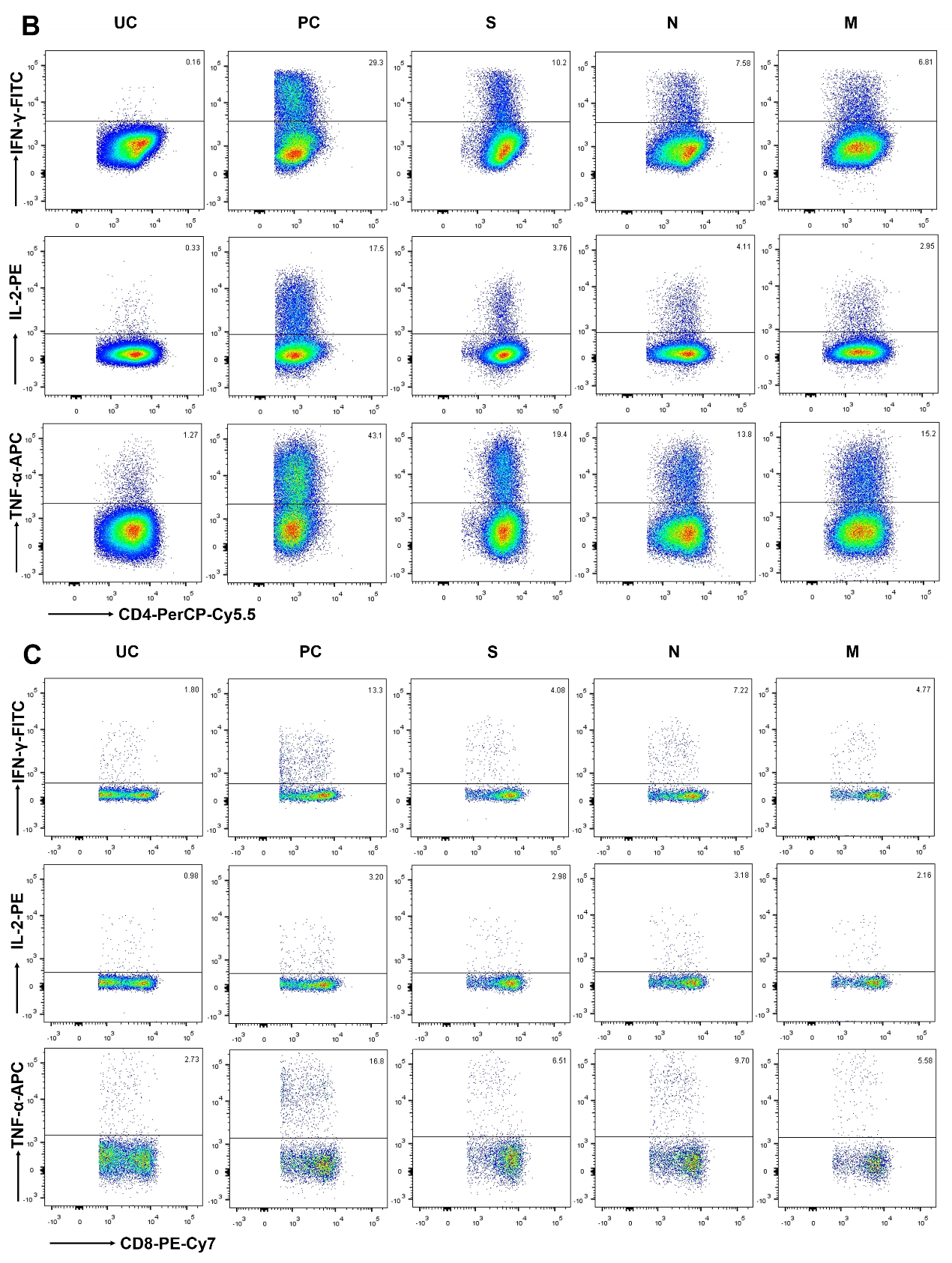
Figure S1. Characterization of T cell responses specific to SARS-CoV-2 viral proteins in vaccinees.** (A) Exemplary strategy for gating CD4+ and CD8+ T cells by flow cytometry. Exemplary gating strategy for analyzing CD4+ (B) and CD8+ (C) T cell responses specific to SARS-CoV-2 S, N, and M in vaccinees. UC: unstimulated control; PC: positive control stimulation.


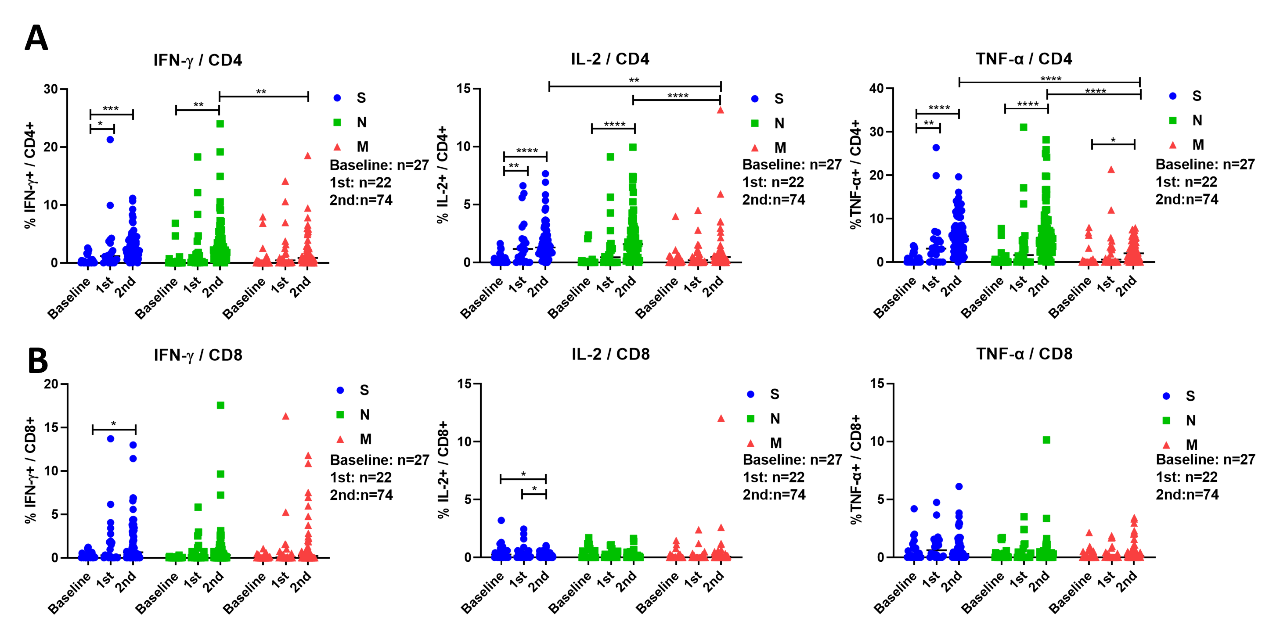


**Figure S2. IFN-γ, IL-2, or TNF-α responses in CD4+ and CD8+ T cells of individuals before and after vaccination.** The frequency of IFN-γ, IL-2, or TNF-α in CD4+ (A) and CD8+ (B) T cells against S, N, and M of SARS-CoV-2 in the participants pre- and post-vaccination. Baseline: pre-vaccination; 1st: first vaccination; 2nd: second vaccination. Each symbol represents an individual donor with a line indicating the median of each group. One-way ANOVA followed by Turkey’s multiple comparisons test was used to test the statistical significance. *,*P*<0.05; **, *P* <0.01; ***, *P* <0.001; ****, *P* <0.0001.


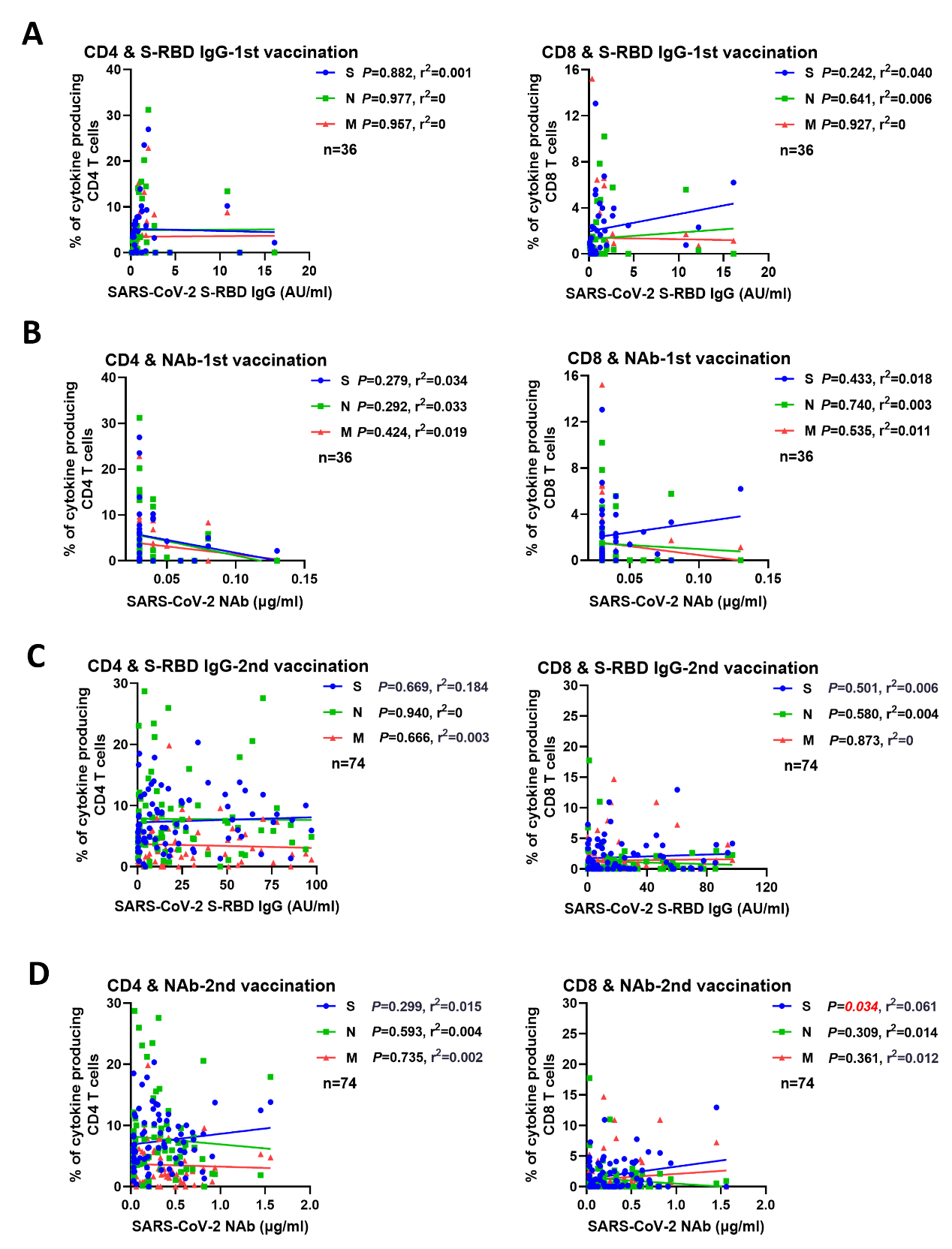


**Figure S3. Correlation between the concentration of SARS-CoV-2 S-RBD IgG and NAb and the magnitude of SARS-CoV-2-specific T cell responses.** The correlation between the magnitude of CD4+ and CD8+ T cell responses specific to S, N, or M and SARS-CoV-2 S-RBD IgG (A) and NAb (B) levels after the first vaccination are shown. The correlation between the magnitude of CD4+ and CD8+ T cell responses specific to S, N, or M and SARS-CoV-2 S-RBD IgG (C) and NAb (D) levels after the second vaccination are shown. Pearson product-moment correlation coefficient test was used to test the significance and P value and r^2^ value (correlation coefficient) are indicated in each panel.


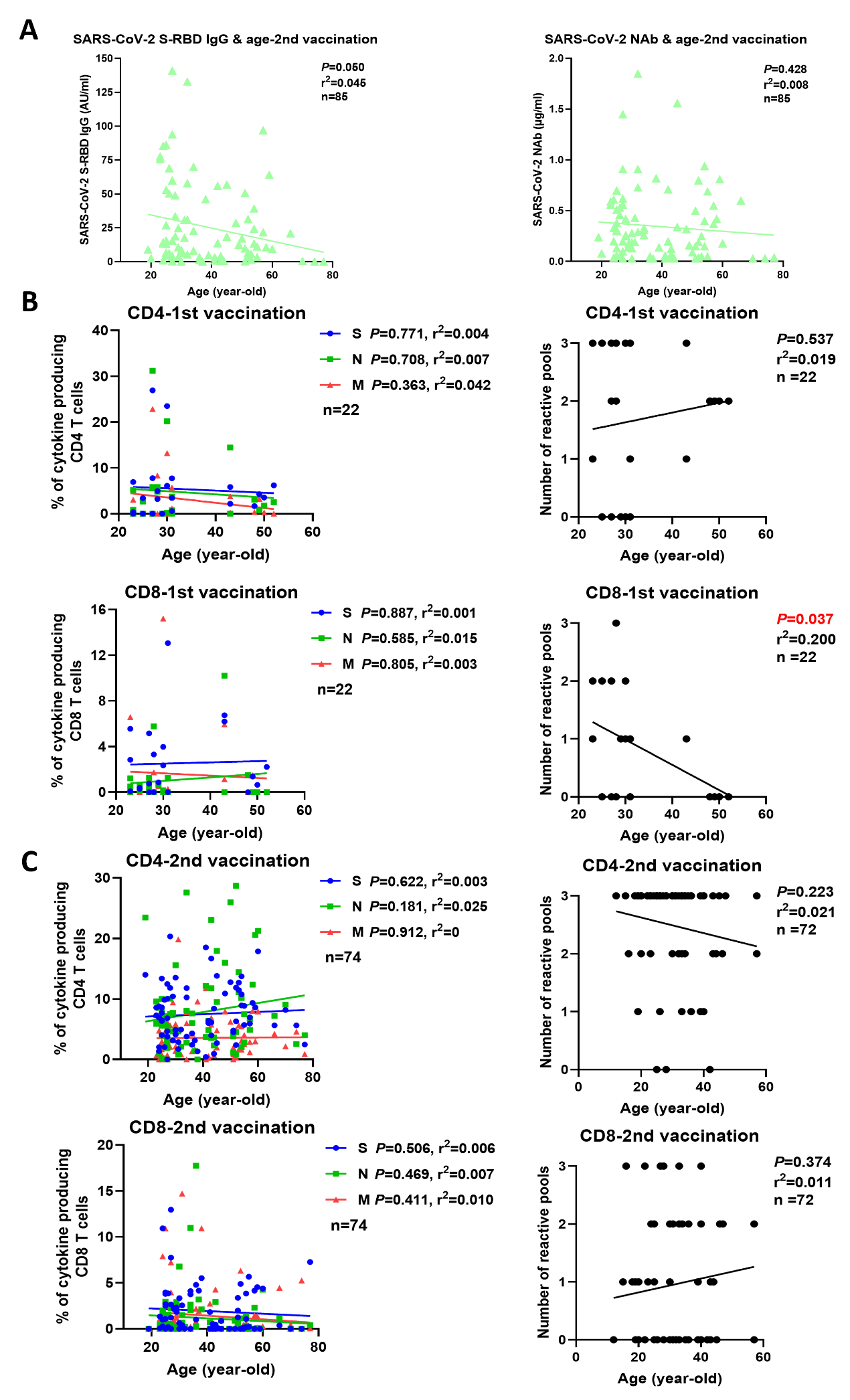


**Figure. S4. Correlation between SARS-CoV-2-specific humoral and cellular immune responses and age.** (A) The correlation between the SARS-CoV-2 S-RBD IgG and NAb levels in plasma and age. (B) The correlation between the magnitude and breadth of SARS-CoV-2-specific CD4+ and CD8+ T cell responses and age after the first vaccination. (C) The correlation between the magnitude and breadth of SARS-CoV-2-specific CD4+ and CD8+ T cell responses and age after the second vaccination. Pearson product-moment correlation coefficient test was used to test the significance and P value and r^2^ value (correlation coefficient) are indicated in each panel.

**
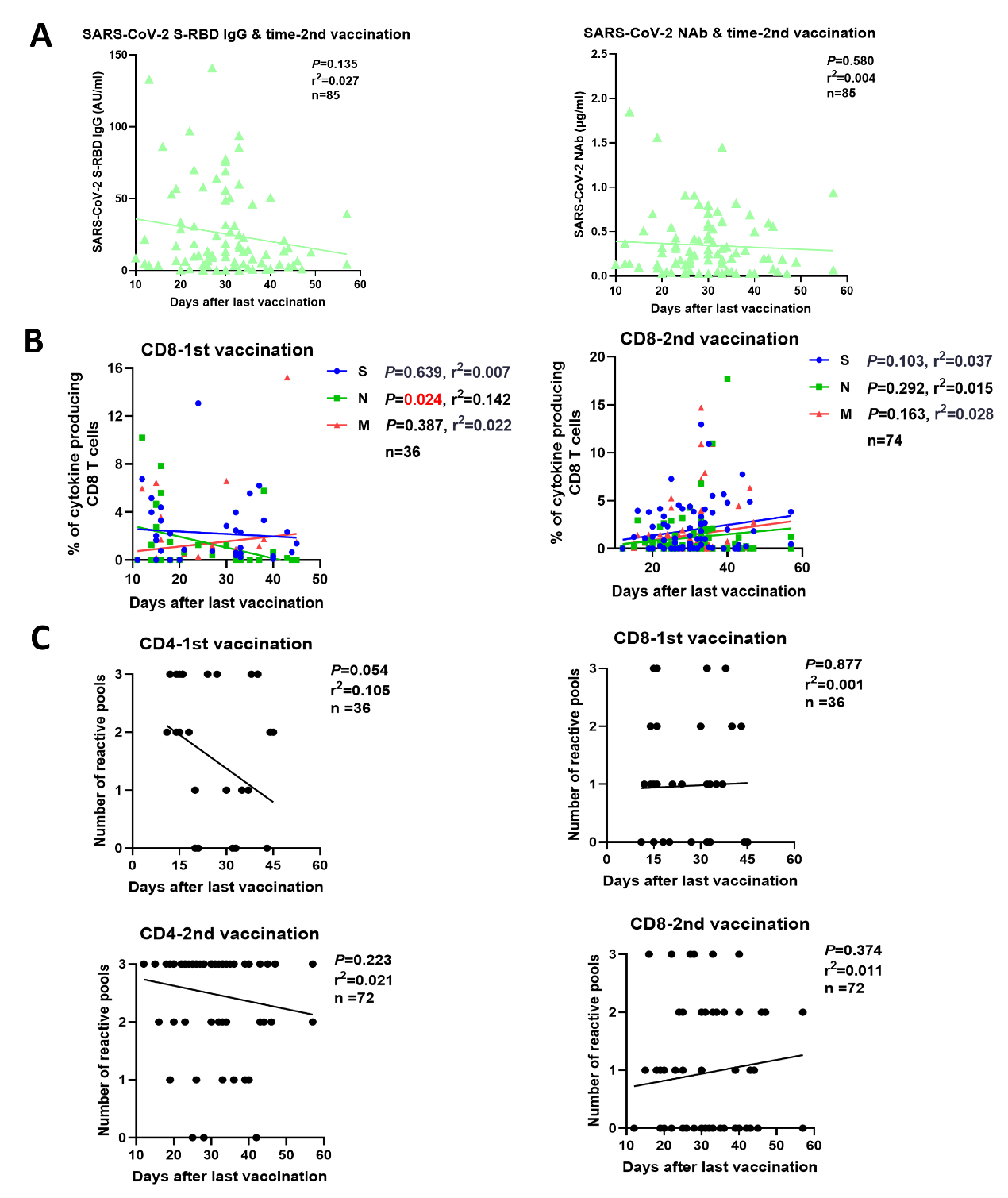
**

**Figure S5. Correlation between the SARS-CoV-2-specific humoral or T cell responses and the time that had elapsed from the last vaccination.** (A) The correlations between SARS-CoV-2 S-RBD IgG and NAb and days after the second vaccination. (B) Correlation between SARS-CoV-2-specific CD8+ T cell responses and days after the first and second vaccination. (C) The correlation between the breadth of SARS-CoV-2-specific CD4+ and CD8+ T cell responses and days after the first and second vaccination. Pearson product-moment correlation coefficient test was used to test the significance and P value and r^2^ value (correlation coefficient) are indicated in each panel.


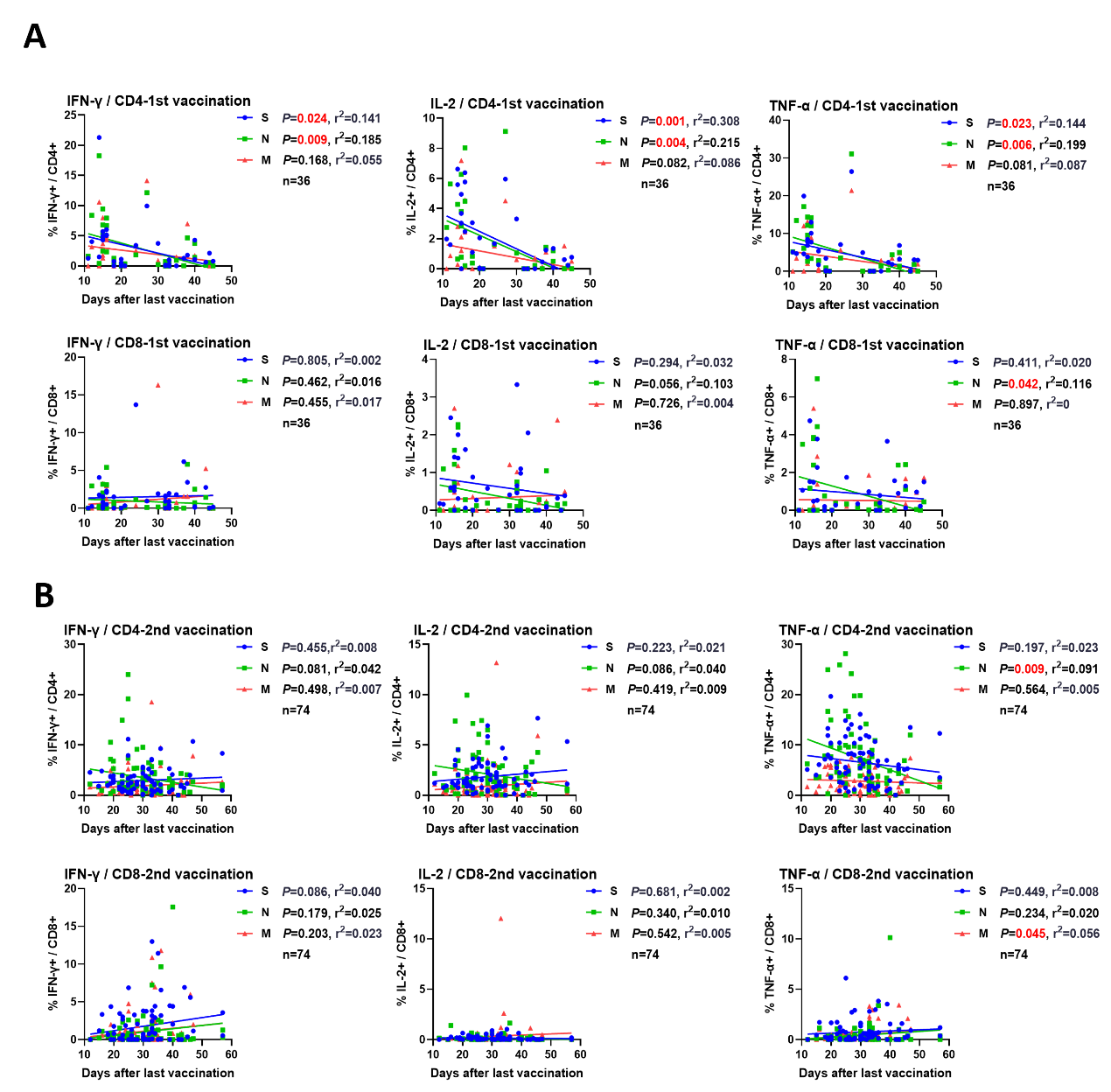


**Figure S6. IFN-γ, IL-2, or TNF-α responses in CD4+ and CD8+ T cells of individuals before and after vaccination.** The frequency of IFN-γ, IL-2, or TNF-α in CD4+ (A) and CD8+ (B) T cells against S, N, and M of SARS-CoV-2 in the participants after the first and second vaccination. Pearson product-moment correlation coefficient test was used to test the significance and P value and r^2^ value (correlation coefficient) are indicated in each panel.


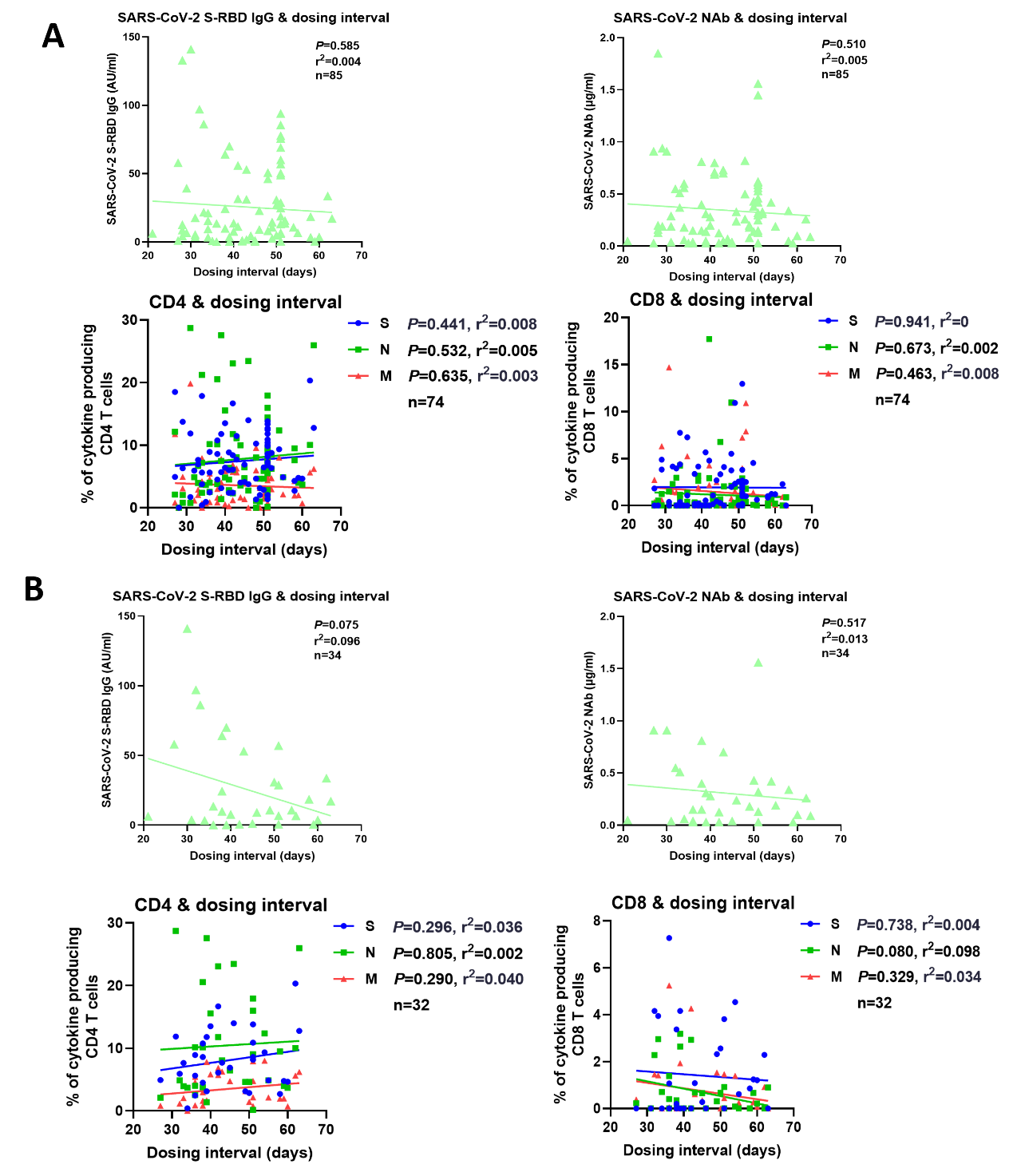


**Figure S7. Correlation between SARS-CoV-2-specific humoral and cellular immune responses and dosing interval.** (A) The correlation between SARS-CoV-2-specific humoral and cellular immune responses and dosing interval in all individuals who received two doses of vaccine. (B) The correlation between SARS-CoV-2-specific humoral and cellular immune responses and dosing interval in 2- to 4-week group. Pearson product-moment correlation coefficient test was used to test the significance and P value and r^2^ value (correlation coefficient) are indicated in each panel.
